# Ventilatory changes during the use of heat and moisture exchangers in patients submitted to mechanical ventilation with support pressure and adjustments in ventilation parameters to compensate for these possible changes: a self-controlled intervention study in humans

**DOI:** 10.5935/0103-507X.20170026

**Published:** 2017

**Authors:** Jeanette Janaina Jaber Lucato, Thiago Marraccini Nogueira da Cunha, Aline Mela dos Reis, Patricia Salerno de Almeida Picanço, Renata Cléia Claudino Barbosa, Joyce Liberali, Renato Fraga Righetti

**Affiliations:** 1 Curso de Fisioterapia, Centro Universitário São Camilo - São Paulo (SP), Brasil.; 2 Departamento de Pacientes Graves, Hospital Israelita Albert Einstein - São Paulo (SP), Brasil.; 3 Serviço de Reabilitação, Hospital Sírio-Libanês - São Paulo (SP), Brasil.

**Keywords:** Respiration, artificial, Humidifiers, Ventilator weaning, Temperature, Intensive care units

## Abstract

**Objective:**

To evaluate the possible changes in tidal volume, minute volume and respiratory rate caused by the use of a heat and moisture exchanger in patients receiving pressure support mechanical ventilation and to quantify the variation in pressure support required to compensate for the effect caused by the heat and moisture exchanger.

**Methods:**

Patients under invasive mechanical ventilation in pressure support mode were evaluated using heated humidifiers and heat and moisture exchangers. If the volume found using the heat and moisture exchangers was lower than that found with the heated humidifier, an increase in pressure support was initiated during the use of the heat and moisture exchanger until a pressure support value was obtained that enabled the patient to generate a value close to the initial tidal volume obtained with the heated humidifier. The analysis was performed by means of the paired *t* test, and incremental values were expressed as percentages of increase required.

**Results:**

A total of 26 patients were evaluated. The use of heat and moisture exchangers increased the respiratory rate and reduced the tidal and minute volumes compared with the use of the heated humidifier. Patients required a 38.13% increase in pressure support to maintain previous volumes when using the heat and moisture exchanger.

**Conclusion:**

The heat and moisture exchanger changed the tidal and minute volumes and respiratory rate parameters. Pressure support was increased to compensate for these changes.

## Abstract (pt)

**Objetivo:**

Avaliar as possíveis alterações do volume corrente, volume-minuto e frequência respiratória causadas pela utilização de trocadores de calor e umidade em pacientes submetidos à ventilação mecânica na modalidade pressão de suporte, e quantificar a variação da pressão de suporte necessária para compensar o efeito causado pelo trocador de calor e umidade.

**Métodos:**

Os pacientes sob ventilação mecânica invasiva na modalidade pressão de suporte foram avaliados utilizando umidificadores aquecidos e trocadores de calor e umidade. Caso o volume encontrado com uso de trocadores de calor e umidade fosse menor que o achado com o umidificador aquecido, iniciava-se o aumento da pressão de suporte, perante o uso de trocadores de calor e umidade, até ser encontrado um valor de pressão de suporte que possibilitasse ao paciente gerar um valor próximo do volume corrente inicial com umidificador aquecido. A análise foi realizada por meio do teste *t* pareado, e os valores de incremento foram expressos em porcentagem de aumento necessário.

**Resultados:**

Foram avaliados 26 pacientes. O uso de trocadores de calor e umidade aumentou a frequência respiratória, e reduziu o volume corrente e o volume-minuto, quando comparados com o uso do umidificador aquecido. Com o uso de trocadores de calor e umidade, os pacientes precisaram de um incremento de 38,13% na pressão de suporte para manter os volumes prévios.

**Conclusão:**

O trocador de calor e umidade alterou os parâmetros de volume corrente, volume-minuto e frequência respiratória, sendo necessário um aumento da pressão de suporte para compensar estas alterações.

## INTRODUCTION

During breathing, inspired air is heated and humidified as it passes through the oral, nasal and pharyngeal cavities. When a patient receives ventilatory support through an endotracheal tube, these physiological mechanisms are suppressed,^(1-3)^ as the superior airway does not perform its normal function of warming and humidifying the air due to the presence of the artificial airway, and the medical gases from tanks or central tube systems are completely devoid of moisture.^(4)^ This heating and moisture deficit has been recognized as damaging to the tracheobronchial mucosa and may lead to undesirable clinical repercussions.^(3,5-7)^

The conditioning of inspired gases is essential to promote adequate heating and humidification.^(2)^ The humidification and heating tasks can be performed either actively, by means of heated humidifiers (HHs), or passively, by means of heat and moisture exchangers (HMEs).

Heated humidifiers are widely used because they promote adequate heating and humidification, but they have some drawbacks, such as cost,^(7-14)^ condensation of water vapor in the ventilation circuit and reservoir, potential for bacterial contamination,^(8,15,16)^ need for a power supply^(15)^ and need for a constant water supply.^(9,17,18)^

The use of HMEs has recently increased in an effort to reduce water loss and condensation in the ventilatory circuit. The HME also offers other advantages, such as low cost,^(8-14,19)^ ease of use,^(15)^ microbiological filter^(19-21)^ and no requirement for a power source.^(22)^ These devices are placed between the endotracheal tube and the Y-connector of the patient's ventilator circuit^(2,23-25)^ and essentially retain moisture and heat during expiration and then release them into the inspired dry air, thereby returning heat and moisture to the patient's airways.^(2,9,10,26-29)^

We must be careful when using an HME, as it can add an excessive resistive load in critical and debilitated patients, especially when high flow is associated with prolonged use. The additional respiratory load imposed by an HME may be substantial for these patients, causing respiratory muscle fatigue and consequent ventilatory failure, or it may affect weaning.^(22)^

Heat and moisture exchangers may cause clinical problems that contraindicate their use. Complications associated with their use include increased resistance,^(22,30-34)^ increased respiratory work,^(18,31,32,35-38)^ and hypercapnia due to the increase in dead space.^(27,32,33,35,39-43)^

The determinants of resistive pressure in mechanically ventilated patients include not only patient airway resistance and inspiratory flow but also the resistance of parts of the ventilator, the endotracheal tube and the HME.^(31)^ Heat and moisture exchangers with a large dead space may have a negative effect on the respiratory function of spontaneously breathing patients due to the increased respiratory work required and may lead to carbon dioxide (CO_2_) retention in paralyzed patients.^(43)^

The direct clinical effects of these drawbacks can be seen, for example, during mechanical ventilator weaning, when, if the addition of dead space changes the alveolar ventilation, the efficiency of spontaneous ventilation may be impaired,^(21,39)^ thus affecting the weaning process. These adverse effects of the addition of HME-related dead space may be even more pronounced in patients who already have low tidal volume (TV) and/or high partial carbon dioxide pressure (PaCO_2_).^(40)^

Changes caused to minute volume should be taken into account in the case of weaning difficulty when an HME is used on patients in spontaneous ventilation, and care should be taken, as we often have to increase the ventilatory parameters to compensate for the presence of an HME. These increases in pressure and volume raise the risk of barotrauma and volutrauma in patients who have more severe changes in respiratory mechanics.^(44)^

We have not identified any studies that quantify the increase in pressure support needed to minimize the adverse effects of HMEs. Therefore, we aim to evaluate the possible changes in TV, minute volume and respiratory rate caused by the use of HMEs in patients submitted to mechanical ventilation in pressure support mode and to evaluate the variation in pressure support required to compensate for the effect caused by the HME's dead space.

## METHODS

This field-based prospective, self-controlled, quantitative analysis was approved by the Research Ethics Committee (protocol 43,222) and was conducted in the adult intensive care unit (ICU) of *Hospital Geral de Carapicuíba* in the state of São Paulo. A total of 26 patients, of both genders, were evaluated. Patients submitted to invasive mechanical ventilation via an endotracheal cannula or tracheostomy were included who were ventilated under pressure support ventilatory mode and who had hemodynamic stability. Exclusion criteria were presence of psychomotor agitation of any origin; use of sedatives; need for positive end-expiratory pressure (PEEP) greater than or equal to 15cmH_2_O; hydroelectrolytic or metabolic changes that might interfere with the patient's respiratory rhythm; pathological respiratory rhythms; bronchopleural fistula (characterized by pleural drainage bubbling) and bronchial hyperreactivity. Minute volume, TV and respiratory rate were measured in two situations: in the first, the patients received gas humidification and heating by HH; in the second, they received it by HME - always with the use of the intermediary that accompanies these devices.

The measurements were performed after 30 minutes of physiotherapeutic treatment, which occurred daily in the ICU and consisted of bronchial hygiene maneuvers, aspiration and positioning in dorsal decubitus elevated to 45°, mainly to avoid the effect of the presence of secretions on the values found during data collection.

Before measuring the desired data, all ventilatory parameters were recorded, with each of the humidification and heating systems used, in addition to blood gases, heart rate, blood pressure and oxygen saturation.

The protocol consisted initially of the use of an HH followed by the use of an HME (Hygrobac S, Tyco, Italy) with trachea (15cm intermediary) in the same patient. The HME used at the time of the measurements was new, weighing 30g, with a dead space of 45mL and a resistance of 2.5cmH_2_O/L and was recommended for TVs greater than 150mL. The HH was connected and installed before the patient's inspiratory branch, and the intermediary used was positioned between the endotracheal cannula and the Y of the ventilatory circuit. The HME was positioned between the Y of the ventilatory circuit and the endotracheal cannula, and, as accompanied by the intermediary, the latter was positioned between the HME and the endotracheal cannula.

At every change of humidification device, 5 minutes were allowed to pass before measurements were taken. The TV and minute volume measurements were taken using a ventilometer (Ferraris Mark 8, England) on the exhalation valve for 1 minute, when the respiratory rate was also verified. The patient was excluded from the study if the ventilator presented bias flow with flow sensitivity that did not allow its deactivation.

After the first measurement using the HH, the values were annotated and later served as a parameter for continuity of the study measurements. If the TV found using the HME was lower than the HH finding, the other part of the study was started, which entailed increasing the pressure support by 1 in 1cmH_2_O with the use of the HME until a pressure support value was found that would allow the patient to generate a TV value with a difference of less than 10% of the initial value found with the HH. Respiratory compensations (such as increased respiratory rate) shown by the patient were also observed. At this stage, with each increase of 1cmH_2_O in pressure support, 5 minutes were allowed to pass before measurements were taken.

At the end of data collection, the patients were returned to the device for conditioning of inspired gases and to ventilatory parameters that were being used prior to the study.

### Data analysis

The mechanical ventilation time data are expressed as medians and interquartile ranges, and the other numeric data are expressed as means and standard errors. Data were tested for normality using the Shapiro-Wilk test. The comparisons between groups using an HH and an HME in the minute volume, TV, respiratory rate and pressure support evaluations were performed using the paired *t* test. The SigmaStat 11.0 statistical package for Windows was used, and a p value of < 0.05 was adopted for statistical significance.

## RESULTS

Twenty-six patients in the general ICU of a large hospital in São Paulo were selected. The patients' clinical characteristics are shown in table 1.

**Table 1:** Patient clinical characteristics

Variables	
Age (years)	62.36 ± 12.64
Gender (f:m)	12:14
Mechanical ventilation time (days)*	8 (5.5 - 16.5)
Blood pressure	
Systolic (mmHg)	127.18 ± 18.4
Diastolic (mmHg)	81.72 ± 22.0
Blood gases	
pH	7.43 ± 0.07
PaCO_2_ (mmHg)	36.8 ± 4.43
PaO_2_ (mmHg)	98.3 ± 19.8
HCO_3_ (mEq/L)	24.88 ± 5.012
SaO_2_ (%)	97.1 ± 2.08
Ventilatory parameters	
Support pressure (cmH_2_O)	11.94 ± 2.99
PEEP (cmH_2_O)	6.8 ± 1.32
Peak inspiratory pressure (cmH_2_O)	18.44 ± 3.53
First diagnosis upon ICU admission	
COPD	11
Sepsis	9
Acute myocardial infarction	4
Ischemic stroke	1
Pneumonia	1

f - female; m - male; PaCO_2_ - partial carbon dioxide pressure; PaO_2_ - partial oxygen pressure; HCO_3_ - bicarbonate; SaO_2_ - arterial oxygen saturation; PEEP - positive end-expiratory pressure; ICU - intensive care unit; COPD - chronic obstructive pulmonary disease.

* Median and interquartile range. Results are expressed as means ± standard deviations, medians (25% - 75%).

**Figure 1:**
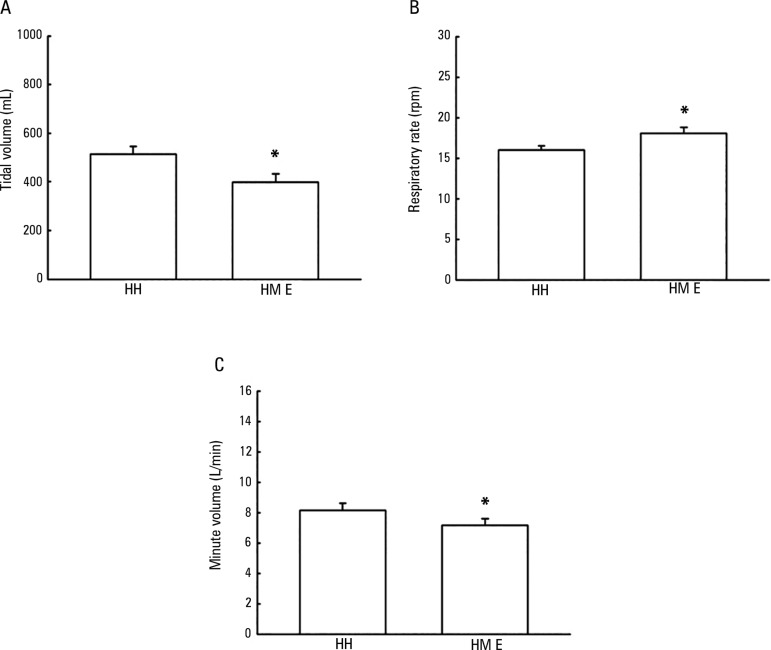
Tidal volume (A), respiratory rate (B) and minute volume (C) values when evaluating patients using heated humidifier and heat and moisture exchanger devices. HH - heated humidifier; HME - heat and moisture exchanger. * p < 0.001, when compared with the use of a heated humidifier.

Figure 1A shows the TV values with the use of the HH and the HME. There was a decrease in patient TV when using the HME (398.3 ± 35.3mL) compared with the HH (514.1 ± 32.2mL), showing a difference of 115.8 ± 14.5mL between the use of the two devices (p < 0.001).

Figure 1B shows the respiratory rate values with the use of the HH and the HME. There was an increase in patient respiratory rate when using the HME device (18.0 ± 0.7rpm) compared with the HH (16.0 ± 0.5rpm), showing a difference of 2.03 ± 0.4rpm between the use of the two devices (p < 0.001).

Figure 1C shows the minute volume values using the HH and the HME. There was a decrease in patient minute volume when using the HME device (7.14 ± 0.4L/minute) compared with the HH device (8.14 ± 0.4L/minute), showing a difference of 1.0 ± 0.2L/minute between the use of the two devices (p < 0.001).

**Figure 2:**
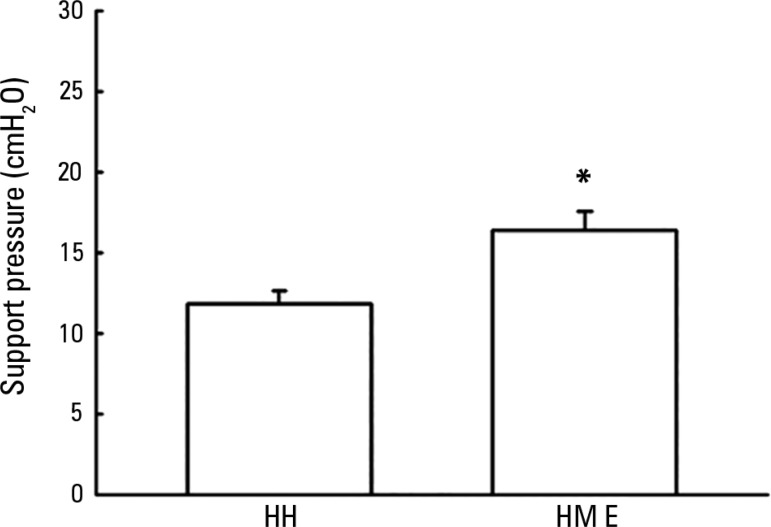
Support pressure values necessary to maintain baseline tidal flow with the use of a heated humidifier in the evaluation of patients using a heat and moisture exchanger. HH - heated humidifier; HME - heat and moisture exchanger. * p < 0.001, when compared with the use of a heated humidifier.

Figure 2 shows the pressure support adjustment values for maintaining baseline TV using the HH when evaluated using the HME. There was a need for increased pressure support when using the HME device (16.3 ± 1.2cmH_2_O) when compared with the HH device (11.8 ± 0.7cmH_2_O) to maintain the baseline TV. An increase of 4.5 ± 0.7cmH_2_O of pressure support was required - approximately 38.13% of the baseline value (p < 0.001).

## DISCUSSION

In mechanically ventilated patients, the humidification device used is occasionally ignored when determining respiratory system mechanics, which can lead to unnecessary treatment and can be responsible for slowing the transition to spontaneous breathing. We evaluated changes to the TV, minute volume and respiratory rate of patients with artificial airways, ventilated in pressure support mode and receiving humidification and heating by HMEs. Our goals were to know whether the HME affected the TV, minute volume and respiratory rate measurements when inserted between the endotracheal tube and the mechanical ventilator circuit and to establish by how much would we need to increase the pressure support to reach the initial values of these parameters. Thus, we needed to ascertain the values without the use of the HME. To accomplish this goal without leaving the patient with no humidification and heating system, the initial measurement was taken with the HH as the baseline. Our study showed that when using HMEs in these patients, an increase of 38.13% from the baseline pressure support was required.

In an earlier study, an analog respiratory system was constructed using a mechanical model to simulate normal- and high-demand situations. Three different levels of inspiratory effort were simulated to calculate the resistance imposed by the HME. The measurements were obtained with dry and saturated HMEs. Resistance increased with HME saturation but showed little increase in response to increased flow.^(34)^

In a systematic review to identify the best humidification system for spontaneously breathing tracheostomized patients, Wong et al.^(45)^ found the HME to be the preferred humidification option, as it reduced pulmonary complications and improved patient collaboration.

Lucato et al.^(46)^ recently conducted a study in which they evaluated the vital capacity and maximum inspiratory pressure of 20 healthy young adults in two situations: with and without an HME coupled to a ventilometer or pressure manometer. The use of the HME did not modify either pulmonary volumes or respiratory muscle strength and could be used safely with these devices to reduce the occurrence of pulmonary infection.

Although we did not find a significant increase in resistance in our previous studies^(34)^ using the HME, nor any changes in lung volume and muscle strength,^(46)^ other studies have shown that resistance to gas flow during use of the HME increased with increasing material density^(10,20)^ and may also increase with increasing flow^(22,47)^ and duration of use.^(22,30,31,34,48,49)^ This increase in resistance in the ventilatory circuit may lead to an incorrect evaluation of respiratory system mechanics, inappropriate treatment (bronchodilators) or difficulty in mechanical ventilation weaning,^(31)^ in addition to increasing the patient's breathing effort.^(18,31,50)^

The use of an HME causes an increase in dead space of an amount equal to its internal volume. The patient should increase their respiratory rate, TV or both to maintain normal alveolar ventilation in the presence of increased dead space related to the use of the HME.^(10,21,43)^ When patients were able to increase their respiratory rate and TV, arterial CO_2_ remained constant. When a patient was unable to increase their minute volume due to either respiratory muscle weakness or fatigue or paralysis, the CO_2_ concentration increased.^(10,33,35,43)^

In the current study, 26 patients were evaluated in regard to changes in TV, minute volume and respiratory rate using an HH and an HME. There was a decrease in TV in patients when using the HME compared with the HH. Corroborating our study, Siqueira et al.^(51)^ subjected 31 neurocritical patients to ventilation, providing two forms of humidification (HME and HH) in a random manner. TV, peak inspiratory and expiratory flow, static compliance and respiratory system dynamics and resistance were evaluated. The HME promoted reductions in TV, peak inspiratory and expiratory flow and dynamic compliance, in addition to increased respiratory system resistance.

In our study, an increase in respiratory rate and a decrease in minute volumes were observed in patients using the HME device compared with using the HH. However, when Boyer et al.^(52)^ evaluated the effects of the HME and the HH on respiratory rate, minute volume, carbon dioxide concentration (ETCO_2_), oxygen saturation, airway occlusion pressure (P0.1) and perception of comfort during non-invasive mechanical ventilation (NIMV), they found no differences between the HH and the HME in any of the parameters studied. The increase in respiratory rate of the patients in our study was not enough to maintain minute volume, which decreased due to the significant reduction in TV.

Jaber et al.'s study,^(40)^ also using NIMV, concluded that during NIMV, increased dead space may adversely affect ventilatory function and gas exchange. The use of the HME may lead to a significant increase in PaCO_2_, despite a significant increase in minute volume. In this study, respiratory effort was not measured, but P0.1 increased significantly when the HME was added into the ventilatory circuit, suggesting that the device may alter the efficiency of NIMV in some patients, especially in the very debilitated. An increase in minute volume resulting from the additional dead space and an increase in P0.1 can lead to an overload of the respiratory muscles. Considering the aforementioned studies, the increased respiratory rate of patients in the present study was not sufficient to maintain minute volume because there was a decrease in TV, likely due to a response to the increased resistance imposed by the device.

Girault et al.^(21)^ also observed that in patients with chronic respiratory failure, the airway humidification type may negatively affect mechanical ventilation efficacy. They evaluated the performance of the HH and the HME in regard to diaphragmatic muscle activity, respiratory pattern, gas exchange and respiratory comfort during mechanical ventilation weaning using pressure support ventilation. Their results revealed that the HME significantly increased all respiratory effort variables (inspiratory respiratory effort, time pressure product, changes in esophageal and transdiaphragmatic pressure and dynamic intrinsic PEEP) and also produced an increase in PaCO_2_, which was insufficiently compensated for by the increase in minute volume. These effects were counterbalanced by an increase in pressure support level, corroborating our study, which demonstrated that increased pressure support was needed when using an HME device (16.3 ± 1.2cmH_2_O) compared to HH (11.8 ± 0.7cmH_2_O) to maintain baseline TV. Therefore, there was a need for an increase of 4.5 ± 0.7cmH_2_O in pressure support, which corresponds to approximately 38.13% of the baseline value.

The direct clinical impact of the use of the HME can be seen during mechanical ventilator weaning. If the addition of dead space changes alveolar ventilation, the efficiency of spontaneous ventilation may be impaired, thus affecting the weaning process.

It is important to take into account not only the endotracheal tube and the mechanical ventilator but also the additional workload imposed by an HME when the patient is in the weaning process.

The non-evaluation of the temperature gradient between the ambient and tracheal air of the evaluated patients is a limitation of the present study, as this gradient could have changed the humidification and heating performance of the HME, or even respiratory system resistance. Roustan et al.^(53)^ noted that the performance of a hydrophobic HME depends on the ambient temperature because a high ambient temperature reduces the thermal gradient between the two sides of the HME. In this regard, Thomachot et al.^(54)^ evaluated ten sedated patients who were ventilated for three consecutive 24-hour periods with a heated humidifier, a hydrophobic HME and a hygroscopic HME and showed that tracheal temperature measurements revealed no differences in ambient air temperature. The increased resistance in this regard appears to be related to high temperatures raising the moisture and occlusion of a device in prolonged use. In view of this likelihood and despite this limitation, we emphasize that all patients were evaluated in the same period with the use of the two devices and at the same room temperature. We also emphasize that the device used was new at the time of the evaluation to avoid the effects of HME occlusion or saturation.

Another limitation of our study was that we adopted the protocol, used in other studies, of waiting 5 minutes before taking measurements so that sensory adaptation of the respiratory center to oxygen and CO_2_ could take place.^(55,56)^ It should be borne in mind that the patient is taken from a condition of pressure and volumetric equilibrium to a transitional condition, and we do not know the time necessary for the new equilibrium to be established. Therefore, it is possible that 5 minutes of evaluation is not sufficient time to stabilize the ventilatory adjustments required in this type of situation.

## CONCLUSION

The use of an heat and moisture exchanger changed the tidal volume, minute volume and respiratory rate parameters. Increased pressure support was required to compensate for these changes.
